# Feasibility, clinical efficacy, and well-being outcomes of an online singing intervention for postnatal depression in the UK: SHAPER-PNDO, a single-arm clinical trial

**DOI:** 10.1186/s40814-023-01360-9

**Published:** 2023-07-27

**Authors:** Rebecca H. Bind, Kristi Sawyer, Katie Hazelgrove, Lavinia Rebecchini, Celeste Miller, Subeyda Ahmed, Paola Dazzan, Nick Sevdalis, Ioannis Bakolis, Rachel Davis, Maria Baldellou Lopez, Anthony Woods, Nikki Crane, Manonmani Manoharan, Alexandra Burton, Hannah Dye, Tim Osborn, Lorna Greenwood, Rosie Perkins, Daisy Fancourt, Carmine M. Pariante, Carolina Estevao

**Affiliations:** 1 Department of Psychological Medicine, Institute of Psychiatry, Psychology and Neuroscience, King’s College London, 5 Cutcombe Rd, Brixton, London, SE5 9RT UK; 2 Centre for Implementation Science, Health Service and Population Research Department, Institute of Psychiatry, Psychology & Neuroscience, 16 De Crespigny Park, London, SE5 8AB UK; 3 Culture Team, King’s College London, Somerset House East Wing, London, WC2R 2LS Strand UK; 4 South London and Maudsley NHS Foundation Trust, Denmark Hill, London, SE5 8AZ UK; 5 Department of Behavioural Science and Health, University College London, Gower Street, London, WC1E 6BT UK; 6 Breathe Arts Health Research, The Clarence Centre, 6 St George’s Circus, London, SE1 6FE UK; 7 Centre for Performance Science, Royal College of Music, London, UK; 8 Faculty of Medicine, Imperial College London, London, UK

**Keywords:** Art intervention, Online delivery, Postnatal depression, Singing intervention, COVID-19 lockdown

## Abstract

**Background:**

Postnatal depression (PND) affects over 12% of mothers, with numbers rising during COVID-19. Singing groups can support mothers with PND; however, online delivery has never been evaluated. SHAPER-PNDO, a single-arm clinical trial, evaluated the feasibility, clinical efficacy, and well-being outcomes of a 6-week online version of Breathe Melodies for Mums (M4M) singing intervention developed for mothers with PND during COVID-19 lockdowns.

**Methods:**

The primary objective of this study was to assess the feasibility of a group online singing intervention for new mothers with postnatal depression. This was ascertained through recruitment rates, study retention rates, attendance rates to the singing sessions, and study completion rates. The secondary objective of the study was to assess the clinical efficacy and well-being outcomes of the singing intervention. Specifically, we measured change in Edinburgh Postnatal Depression Scale (EPDS), State-Trait Anxiety Inventory (STAI), Perceived Stress Scale (PSS), and Office for National Statistics Wellbeing Scale (ONS) scores from baseline to end-of-intervention (week 6); follow-up assessments were completed at weeks 3, 16, and 32. Mothers were eligible if they scored ≥10 on the baseline EPDS.

**Results:**

Eighty-seven percent of the 37 recruited mothers completed the study, attending, on average, 5 of the 6 group singing sessions. With regard to secondary outcomes, at end-of-treatment, mothers experienced significant reductions in depression (EPDS, 16.6 ± 3.7 to 11.2 ± 5.3, 95% CI [0.79,1.65]), anxiety (STAI-S, 48.4 ± 27.1 to 41.7 ± 26.8, 95% CI [4.96, 17.65]) and stress (PSS, 29.0 ± 5.7 to 19.7 ± 5.3, 95% CI [1.33, 7.07]); and, furthermore, significant improvements in life satisfaction (ONS, 50.5 ± 23.0 to 72.8 ± 11.7, 95% CI [− 39.86, − 4.64]) and feelings of worthwhileness (ONS, 51.7 ± 30.4 to 78.6 ± 15.1, 95% CI [− 52.79, − 0.85]). Reduction on the EPDS correlated with a reduction on the BDI and the STAI-S and maternal childhood maltreatment was predictive of a smaller treatment response.

**Conclusions:**

M4M online was feasible to mothers who partook in the programme. Furthermore, M4M online supports the mental health and well-being of new mothers experiencing PND, especially when barriers to in-person treatment are present.

**Trial registration:**

ClinicalTrials.gov NCT04857593. Registered 22 April 2021, retrospectively registered;

**Supplementary Information:**

The online version contains supplementary material available at 10.1186/s40814-023-01360-9.

## Key messages regarding feasibility

- What uncertainties existed regarding the feasibility?

The feasibility of using an online singing intervention for new mothers with postnatal depression and their babies was previously unknown. In addition, the feasibility of utilising this intervention not only in times of social isolation, such as during a pandemic, but thereafter for mothers with additional barriers to treatment was unknown, in particular, recruitment rates, retention rates, attendance rates, study completion rates, and scalability to power a subsequent RCT.

- What are the key feasibility findings?

We found that while recruitment was lower than anticipated, retention and study completion rates were high. Overall, 37 mothers and their babies were recruited into the study and attended 5 out of 6 (83%) prescribed group singing sessions. Of the 37 mothers, 32 (87%) remained in the study and completed the end-of-intervention visit at week 6. Additionally, 21 (57%) completed the follow-up assessment at week 32. Of note is that only one participant formally withdrew from the study.

- What are the implications of the feasibility findings for the design of the main study?

For the main study, we will need to recruit study participants from many more channels than was possible for this pilot study in order to achieve a sufficient sample size. Despite this, the findings from this study also imply that our intervention is feasible, given our singing session attendance rates, study retention throughout the intervention, and study completion rates up to 6 months post-intervention.

## Background 

### Postnatal depression and treatment

Postnatal depression (PND) is underreported, but estimated to affect at least 12% of new mothers [1]. Symptoms may include low mood, anhedonia, insomnia, changes in appetite, fatigue, and, in the most severe of cases, suicidality [2]. Moreover, suicide remains the leading cause of maternal mortality across the first postnatal year [3]. The effects of PND carry implications for maternal outcomes and the developing relationship between the mother and the infant [4], and, if left untreated, PND has been linked with suboptimal offspring developmental outcomes [5], altogether strongly underscoring the necessity for treatment.

There are still many barriers to treatment in the perinatal period at both the system and individual levels [6]: while psychological therapy has shown positive results in primary care [7], there are logistical, cultural, and financial obstacles to treatment, including inadequate resources in place to routinely screen and identify symptoms, especially cross-culturally, as well as long waiting lists and scarcity of professionals, especially outside urban areas [6]; likewise, anti-depressants are shown to be moderately effective in inducing response, remission, and reduction of depressive symptoms [8], but their uptake and adherence is low in the perinatal population, especially among breastfeeding mothers [9, 10]. Furthermore, mothers with PND may face additional barriers, including lack of symptom insight and of service awareness, the pressure of societal stigma, the fear of separation from their infant [6], medical and psychiatric comorbidities, and socioeconomic difficulties.

### The impact of the COVID-19 pandemic on perinatal depression

The COVID-19 pandemic created unique challenges for new mothers, rendering them at greater risk of PND [11]. Home isolation and mandatory physical distancing, which were ubiquitously experienced worldwide at different stages of the pandemic in 2020–2022, resulted in loneliness, isolation, financial stress, decreased social support (including reduced mother–baby groups and community classes), and increased rates of domestic violence [12]. In fact, self-reported depression and anxiety scores in pregnancy and the postpartum have been significantly higher during the pandemic than in non-pandemic periods [13].

Given the previously-mentioned barriers to treatment, the overall vulnerability that the COVID-19 lockdowns have created in new mothers, and the significant decrease in existing access to care, the need for new and remotely accessible treatment options for mothers with PND has become even more urgent.

### Singing interventions in the postnatal period

Recent publications have highlighted the potential of community-based art interventions for mental and physical well-being [14, 15]. Group singing, in particular, is evidenced to assist recovery in adults with serious mental illness [16] and is associated with increased social bonding [17]. In the UK, new mothers frequently engage in community group activities with their infants. These groups offer relaxation, positive social interaction, and support [18, 19]. Research from us and others shows that singing to infants improves mother–infant interactions and reduces psychological and biological markers of stress [19–21], an effect that has not been demonstrated in other psychosocial interventions geared towards aiding the mother–infant relationship in mothers with PND [22]. Furthermore, online interventions may be a solution for women with PND who face diagnosis stigma or COVID-19 anxiety, while being more scalable and accessible [23].

Breathe Melodies for Mums (M4M) is an evidence-based community singing intervention delivered in Children’s and Family Centres for new mothers with PND. M4M was initially piloted in London in 2015–17 in a three-arm randomised clinical trial (RCT) [24] and data showed that for women with moderate–severe PND (Edinburgh Postnatal Depression Scale; EPDS > 13), M4M resulted in a significant reduction in PND by week 6 of the intervention and that by the end of the intervention (week 10), 73% of the group had an EPDS < 13.

The scale-up of the community M4M is underway as part of the larger SHAPER programme [25], but the COVID-19 pandemic led to the development of an online adaptation of M4M (SHAPER-PNDO), presented here. This was a 6-weekly online intervention adapted from the original community 10-week M4M, as described in the published protocol [26]. To reduce the risk of disengagement by participants that may happen with online interventions, the duration was shortened from the 10 weeks to 6 weeks, as this is the earliest time point that showed significant antidepressant effects in the community M4M study [24].

### Aims and hypotheses

This single-arm feasibility study aimed to evaluate the feasibility, clinical efficacy, and well-being outcomes of 6 weeks of online delivery of M4M (an intervention led by the community interest company, Breathe Arts Health Research; Breathe). The ambition was to develop a remote intervention that can become a mainstream therapeutic tool in times of social isolation and for mothers who regularly face barriers to care.

We hypothesised that the online M4M intervention would (1) effectively recruit, engage, and retain new mothers with PND; (2) reduce symptoms of PND, anxiety, and stress; and (3) improve perceived loneliness, well-being, and social support. Moreover, we aimed to explore sociodemographic and psychological factors impacting treatment response.

## Objectives

In order to assess the feasibility of the M4M online intervention, data was collected according to the following objectives:

### Primary feasibility objective

The primary objective of this study was to assess the feasibility of a group online singing intervention for new mothers with postnatal depression in order to ensure adequate recruitment for a future RCT. We define study feasibility according to the definition used by Proctor et al. (2011) [27] as recruitment rates, attendance rates, retention rates, and study completion rates, in order to inform upon scalability to power a subsequent RCT.

### Secondary objectives

The clinical efficacy of this intervention was assessed through the following secondary objectives:

Primary:

- To assess the effectiveness of online singing for symptoms of postnatal depression

Secondary:

- To assess whether online singing improves further aspects of mental health, including anxiety and stress
- To ascertain whether online singing improves perceived social support and reduces loneliness
- To explore the uptake and involvement in online singing groups

## Methods

### Intervention: breathe melodies for mums online

We modified the original face-to-face intervention for this online study as follows:

- Groups were up to 15 women (in line with the community M4M) to ensure all participants were visible on one screen and thus create a stronger sense of community and connection.
- The intervention was 6 weeks long, based on the face-to-face intervention evidence that by 6 weeks, there is already a significant improvement in depressive symptoms compared with control interventions [24].
- Mothers were connected from week 2 of the intervention to an optional WhatsApp group, to mirror the social interaction component of the in-person community intervention.

Artists were trained centrally by Breathe to ensure that fidelity to the original M4M was maintained. Furthermore, a member of Breathe was present on each Zoom singing session to monitor and ensure the session was being delivered according to protocol. During the singing portion of the sessions, all participants were muted, while the artist was unmuted and playing background melodies, to ensure a group feeling while avoiding broadband-related sound delays and other background noises. Thus, participants could hear themselves, the artist, and the background melody, with pre-recorded voices, that recreated the community M4M setting. Outside of sessions, mothers had access to a weblink with M4M songs, to allow them to sing at home in between sessions and beyond M4M.

### Patient and public involvement

The protocol for the online Melodies for Mums intervention was co-developed with Breathe Arts Health Research (Breathe), a not-for-profit organisation who develop and deliver evidence-based arts programmes to improve health and well-being. Interventions created by Breathe are designed alongside artists, researchers, healthcare staff, and patients, in order to integrate feedback from as many stakeholders as possible. Thus, the in-person Melodies for Mums programme, which was developed and refined together with artists, researchers, and patients, was then adapted into an online intervention by incorporating feedback from stakeholders who had previously taken part in other Breathe online arts interventions.

### Primary feasibility outcomes: feasibility of the intervention

#### Recruitment and study setting

In order to assess the feasibility (primary objective), the following recruitment procedure was applied: mothers were recruited in the community through social media advertising (both paid advertising and organic social media dissemination) and, during times of partial lockdown, through posters and flyers in baby weigh clinics and other community and clinical centres for postnatal mothers and their babies, as well as signposting via health and social care professionals. Given that the delivery of M4M was online, recruitment was UK-wide.

A total of 37 participants were enrolled to the study across a total of 5 cohorts of recruitment, from February 2021 (recruitment of the first cohort) to the end of June 2021 (end of intervention for the last cohort). Mothers were pre-screened by Breathe, and their details were sent to the research team for baseline assessments and enrolment. While in the original protocol, we had estimated recruitment of 120 women over 12 months, rapid changes in the lockdown procedures forced us to stop the recruitment after only 6 months in order to switch back to the face-to-face M4M programme (see “Discussion”).

#### Eligibility criteria

Mothers were eligible for the study if they were aged 18 or older, had a satisfactory understanding of English, had a child between 0 and 9 months old, and had symptoms of PND, defined as a minimum score of 10 on the EPDS.

Potential participants were considered ineligible if they could not give informed consent or access online sessions (i.e., internet connection, laptop or computer availability). Mothers were provided mobile data vouchers to cover data usage during sessions and assessments if needed.

#### Recruitment, retention, and completion rates

The feasibility of Breathe Melodies for Mums online was assessed by recording participant recruitment rates, retention and attrition rates, intervention attendance, and study completion rates. These measures were collected from the five cohorts through the study period. Furthermore, we considered a traffic light system to understand our study’s feasibility, as discussed in previous literature, which sets specific criteria to determine a pilot study’s suitability to progress to a full-scale RCT [28]. More specifically, according to the traffic light system, a pilot study can be considered at the green stage (go to RCT; no issues arose from the pilot), the amber stage (amend; issues with the pilot that are rectifiable in an RCT), or the red stage (stop; issues with the pilot that cannot be fixed).

### Secondary outcomes: clinical and social assessments

Once they consented to the study, mothers underwent an online baseline assessment via Zoom with two researchers before the first M4M online session. A subsequent online assessment was conducted at week 6 to collect end-of-intervention outcomes. Additionally, at the above time points and in weeks 3, 16, and 32, participants completed a set of self-report questionnaires online. The assessment tools used for clinical and social outcomes (discussed below) were chosen based on their validity, their clinical and research utility, their robustness in capturing symptom severity, and the fact that they have been used in research extensively. Study data were collected and managed using REDCap electronic data capture tools [29] hosted at King’s College London.

#### Effectiveness of online singing for symptoms of postnatal depression

##### Demographics

We collected, via Zoom call, the following questionnaires in interview format: baseline demographics on sociodemographic and economic circumstances, maternal and infant health history, and participation in any other mother–baby activities; the Threatening Life Experiences Questionnaire [30], a validated questionnaire that ascertains experience of stressful life events in the perinatal period; the Child Experience of Care and Abuse Questionnaire [31], a validated interview that assesses for childhood experience of physical and sexual abuse, antipathy, and neglect; the Composite Abuse Scale-Pregnancy Version [32], a validated questionnaire that ascertains experience of intimate partner violence; and the Intrusive Life Events Scale [33], a validated questionnaire that asks about distressing life events at any point.

##### Mental health

The EPDS [34] was collected via self-report questionnaires at baseline and weeks 3, 6, 16 and 32 to assess the effectiveness of online singing interventions on symptoms of PND. To clinically assess for a diagnosis of major depressive disorder at baseline or prior to baseline in lifetime, the Structured Clinical Interview for DSM-IV Disorders (SCID-IV) [35] was collected at baseline. Further, the Hamilton Depression Rating Scale (HDRS) [36] was collected at baseline and at the end-of-intervention (week 6) on Zoom (interview format) to assess whether singing improved the severity of depressive symptoms.

In addition, to evaluate whether singing improved further aspects of mental health, well-being, and social support, the following scales were collected at baseline and weeks 3, 6,16, and 32: the Beck Depression Inventory (BDI) [37], the Office for National Statistics Wellbeing Scale (ONS) [38], the State-Trait Anxiety Inventory (STAI) [39], the Perceived Stress Scale (PSS) [40], the UCLA Loneliness Scale [41], the Multidimensional Scale of Perceived Social Support (MSPSS) [42], and the Short General Self-Efficacy Scale (GSE-6) [43].

### CONSORT 2010 flow diagram

**Fig. 1:**
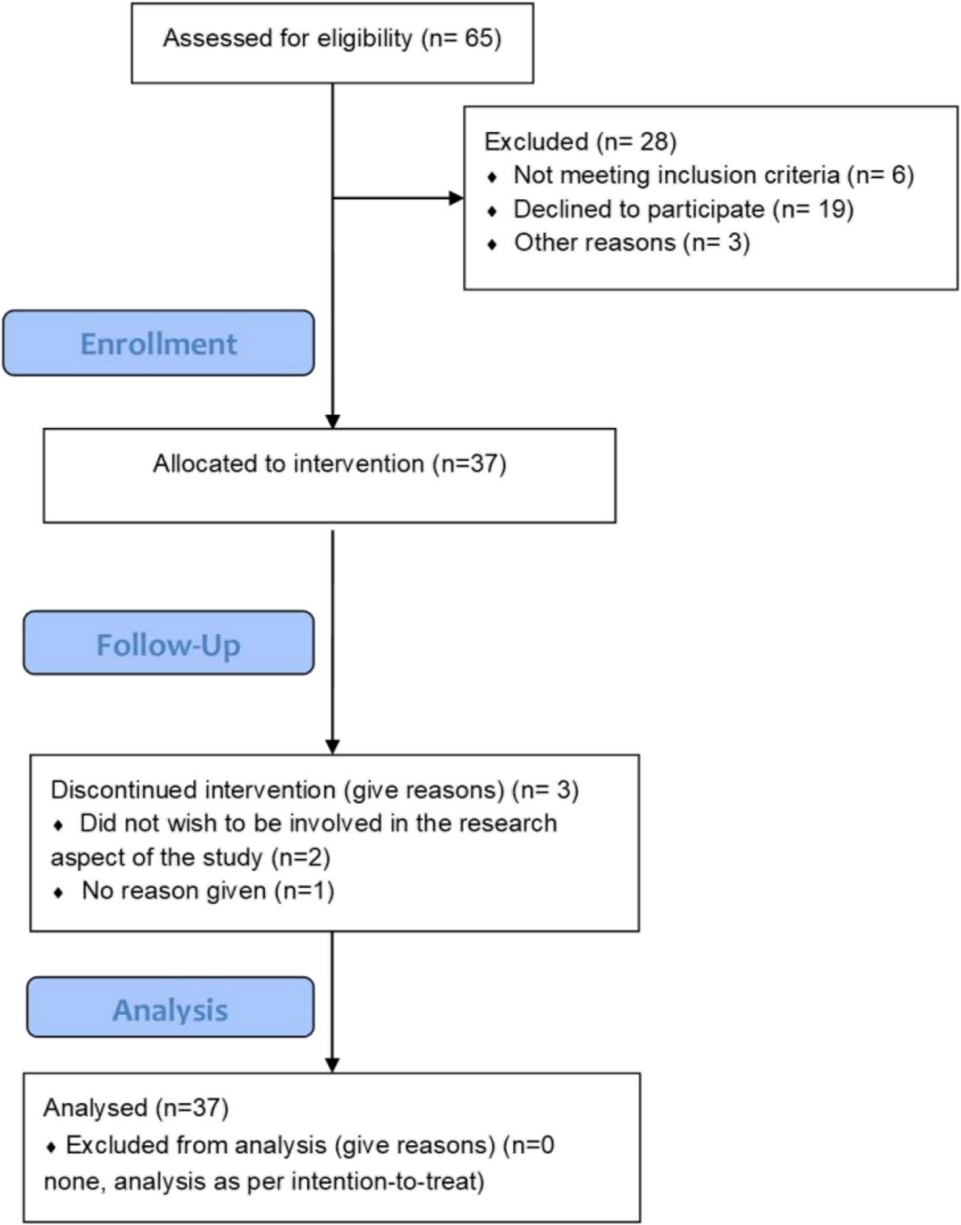
CONSORT 2010 flow diagram for SHAPER-PNDO

This study was a single-arm trial using the following timeline procedure presented in the adapted CONSORT 2010 flow diagram (Fig. 1).

### Statistical analysis

Analyses were conducted with SPSS Statistics version 27 for MacOS (IBM, UK). Before analysis, data were checked for normality. Due to the limited sample size, multiple imputations were not used, and missing values were adjusted to the last observation carried forward.

**Fig. 2:**
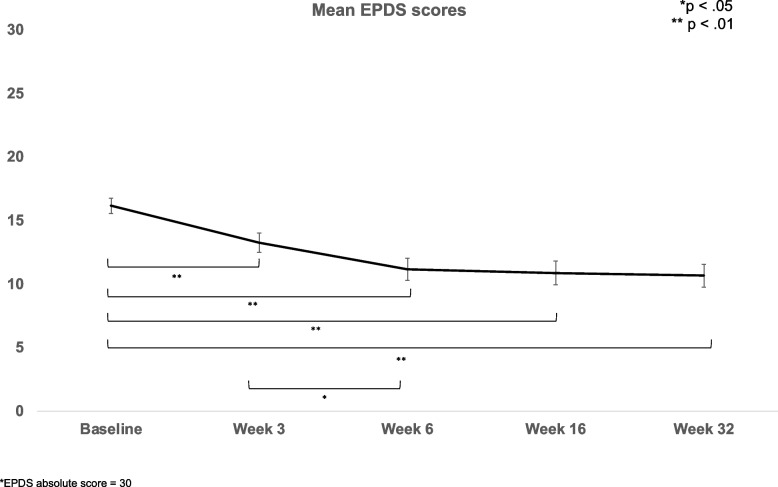
Mean EPDS scores at baseline, week 3, week 6, week 16, and week 32

**Fig. 3:**
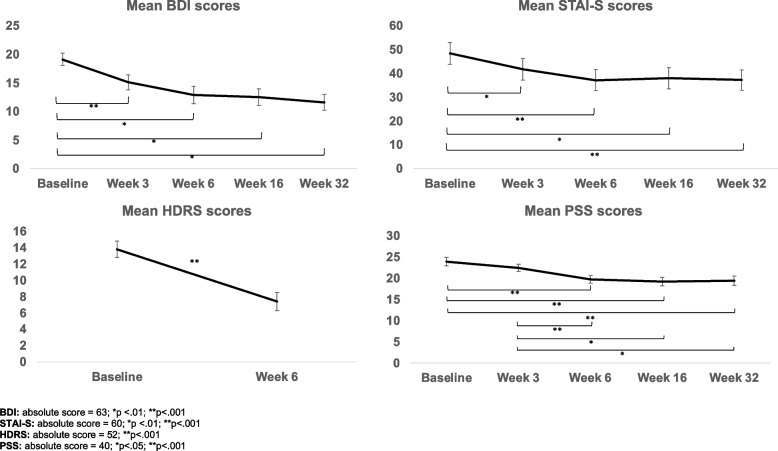
Mean BDI, STAI-S, HDRS, and PSS scores at baseline, week 3, week 6, week 16, and week 32

**Fig. 4:**
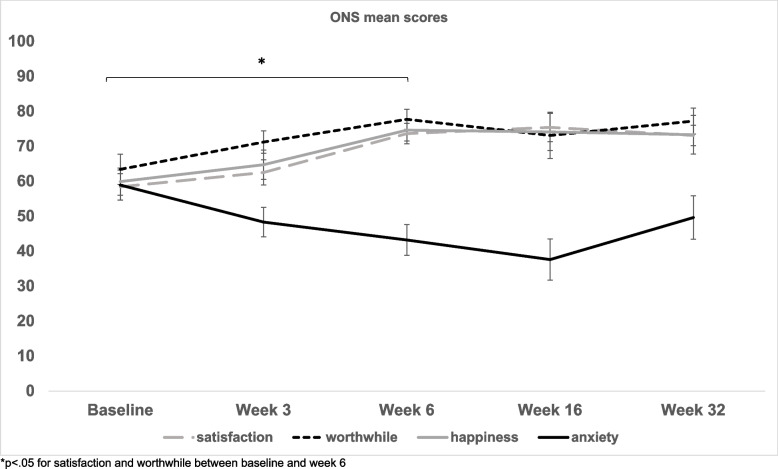
Mean scores on the ONS Wellbeing scale at baseline, week 3, week 6, week 16, and week 32

Our feasibility outcomes (recruitment, retention, attendance, study completion rates), in addition to our demographics, were analysed using frequencies and descriptives. As described in the protocol, our secondary outcome was changed in EPDS between baseline and end-of-intervention, measured using paired-samples *t*-tests and 95% confidence intervals. Additional changes in the aforementioned mental health and well-being scales were analysed using repeated-measures ANOVAs with 95% confidence intervals and Bonferroni corrections for post-hoc comparisons between time points, for tracking changes across all study time points. A fixed-effects regression using data from five time points was run to look for a within-person association between changes in EPDS score and changes in other psychiatric symptoms. Univariate correlations were utilised to examine associations between changes in EPDS score and putative factors affecting treatment response, and relevant variables were included in a hierarchical linear regression model. Means (standard errors) are presented in Figs. 2, 3 and 4.

## Results

### Primary outcome: feasibility of the intervention

Participant recruitment ran from February 2021 to June 2021 and follow-ups for all participants were completed in February 2022. Feasibility was ascertained by recruitment, intervention attendance rate, and study retention rate at each time point. Thirty-seven mothers were recruited into the study (see Fig. 1), and, on average, attended 5 out of 6 (81%) prescribed group singing sessions. Regarding study visits, of the 37 mothers, 32 (87%) remained in the study and completed the end-of-intervention visit at week 6. Additionally, 21 (57%) completed the follow-up assessment at week 32. Regarding online self-report questionnaires, of the 37 participants, 35 (95%) completed the baseline questionnaires, 28 (76%) the week-6 questionnaires, and 18 (49%) the week-32 questionnaires. Of note is that only one participant formally withdrew from the study. Thus, while overall recruitment fell short of the initial target (120 over 12 months), even considering that the recruitment period was halved to 6 months, the retention and completion rates were high, especially if compared with other online interventions (see “Discussion” below).

### Secondary outcomes

#### Clinical and sociodemographic characteristics

The sociodemographic characteristics of the sample at baseline are presented in Table 1. Patient-centred analyses were performed on the 37 participants who completed the intervention. The mean maternal age was 35.3 years old; 34 (92%) mothers were married or cohabiting; 28 (76%) identified as white; 33 (89%) had completed higher education, 32 (87%) were employed or on maternity leave; and 35 (94%) had a household income above £30,000.

**Table 1:** Sociodemographic details and risk factors of SHAPER-PNDO study participants

	*N* = 37	
Maternal age in years, M (SD)	35.3 (4.6)	
Maternal marital status, *n* (%)		
Married/cohabiting	34 (91.9)	
Single with/without a partner	3 (8.1)	
Maternal ethnicity, *n* (%)		
White	28 (75.7)	
Ethnic minority groups	9 (24.3)	
Biological father ethnicity, *n* (%)		
White	26 (70.3)	
Ethnic minority groups	10 (27.0)	
Maternal qualifications, *n* (%)		
Higher education or above	33 (89.2)	
GCSE/A -level or below	4 (10.8)	
Maternal employment status, *n* (%)		
Employed/student/maternity leave	32 (86.5)	
Unemployed/full-time mother	5 (13.5)	
Partner employment status, *n* (%)		
Employed/student	35 (94.6)	
Missing	2 (5.4)	
Household income, *n* (%)		
< £30,000	2 (5.6)	
> £30,000	35 (94.4)	
Infant age in months, M (SD)	4.0 (2.5)	
Infant sex, *n* (%)		
Female	18 (48.6)	
Male	19 (51.4)	
Attending other mother–baby group, *n* (%)		
Yes	17 (45.9)	
No	20 (54.1)	
Childhood experience of abuse, *n* (%)		
Yes	12 (36.4)	
No	21 (63.6)	
Adult experience of intimate partner violence, *n* (%)		
Yes	15 (40.5)	
No	22 (59.5)	
Intrusive life event across lifetime, *n* (%)		
Yes	16 (48.5)	
No	17 (51.5)	
Threatening life event in perinatal period, n (%)		
Yes	18 (52.9)	
No	15 (44.1)	
	Lifetime diagnosis of MDD? *n* (%)	
	No	Yes
Current diagnosis of MDD? *n* (%)		
No	5 (13.5)	10 (27.0)
Yes	4 (10.8)	18 (48.6

With regard to reproductive history, 17 (46%) had previously had a pregnancy that did not result in a live birth (e.g. termination or miscarriage). In terms of birth outcomes: 29 (78%) experienced problems related to their pregnancy or delivery; 22 (60%) required intervention during their delivery, and 19 (51%) of infants were male. At the baseline assessment, the average age of infants was 4 months, and 26 (70%) reported problems with feeding their baby.

##### Childhood maltreatment, adult intimate partner violence, and life events

The prevalence of history of childhood maltreatment, adult intimate partner violence, and life events are presented in Table 1.

Of the 37 mothers, 12 (36%) experienced at least one form of childhood abuse, with 6 (17%) experiencing multiple types. In adulthood, 15 women (41%) reported experiencing intimate partner violence, with 8 (22%) reported experiencing more than one type. Regarding intrusive events, 16 (49%) mothers had experienced an event in their lifetime, and 18 (53%) reported a threatening event during the perinatal period.

##### Mental health

Diagnostic characteristics are presented in Table 1. At baseline, 22 (59%) mothers met clinical criteria for a current major depressive episode, 12 (32%) were currently receiving psychological or talking therapy, and 5 (14%) were on antidepressants. Twenty (54%) mothers had been previously diagnosed by a health professional with a mental health condition (mood or anxiety disorder) and 28 (76%) met clinical criteria on the SCID-IV for a major depressive episode in their lifetime before the baseline assessment. Baseline scores for EPDS and other self-report scales are shown below and in Figs. 2 and 3, as part of the before–after comparisons.

#### Intervention outcomes

##### Primary postnatal depressive symptoms outcome

We first compared total scores on the EPDS between baseline (pre-intervention) and week 6 (end of intervention), as this was the secondary outcome of the study. Mothers experienced a significant reduction in their total EPDS score, from 16.6 ± 3.7 (indicating probable depression) to 11.2 ± 5.3 (possible depression), *t*(36)  = 7.56, *p* < 0.001, 95% CI [0.79,1.65].

Next, to understand the trajectory of depressive symptoms throughout, and following the intervention, we investigated EPDS scores across time. There was a significant effect of time, such that there was a total decline in EPDS scores across the study time points (*F*(4,144) = 24.48, *p* < 0.001; see Fig. 2), and this included significant reductions (Bonferroni corrected *p* < 0.01) from baseline (16.6 ± 3.7, probable depression) to week 3 (mid-intervention, 13.3 ± 4.6, fairly high possibility of depression, 95% CI [1.53, 5.07]), week 6 (end of the intervention, 11.2 ± 5.3, depression possible, 95% CI [3.25, 7.61]), week 16 (follow-up, 10.9 ± 5.6, depression possible, 95% CI [2.99, 8.51]), and week 32 (follow-up, 10.7 ± 5.5, depression possible, 95% CI [3.37. 8.53]). Furthermore, the reduction from baseline to week 3 continued between week 3 and week 6 (Bonferroni corrected, week 6 vs. week 3, *p* = 0.032, 95% CI [0.11, 4.16]). These results indicate that the mothers’ depressive symptoms steadily reduced throughout the intervention to week 6 and that this reduction remained all the way through to 6 months post-intervention (that is, from week 6 to week 32).

##### Secondary postnatal depressive symptoms outcome

We looked at the trajectory of total scores on the BDI and we compared total HDRS scores between baseline (pre-intervention) and week 6 (post-intervention).

Mothers reported significant and sustained reductions in the BDI across the study (*F*(4,132) = 12.32, *p* < 0.001; see Fig. 3). On the BDI, these differences were observed between baseline (18.9 ± 6.6, moderate-severe depression) and week 3 (15.1 ± 7.5, mild-moderate depression, 95% CI [0.62, 7.26]), week 6 (13.0 ± 8.6, mild–moderate depression, 95% CI [1.95, 10.40]), week 16 (12.3 ± 8.4, mild–moderate depression, 95% CI [2.17, 11.0]), and week 32 (11.6 ± 8.1, mild–moderate depression, 95 CI [2.79, 12.09]) (Bonferroni corrected *p* < 0.05 for all comparisons with baseline), with no differences in symptoms between any other time points. This suggests that the largest decrease of symptoms on the BDI occurred immediately within the first 3 weeks of intervention.

On the HDRS, mothers were observed to have a significant decrease in the severity of their depression between baseline (13.8 ± 6.3, indicating mild depression) and week 6 (7.4 ± 6.8, indicating no depression), *t*(36) = 6.81, *p* < 0.001, 95% CI [0.71, 1.55].

##### Further aspects of mental health

To assess whether online singing improved further aspects of mental health, we looked at the trajectory of total scores on the STAI-S and PSS across study time points (see Fig. 3).

Mothers reported significant and sustained reductions in the STAI-S and PSS across the study (*F*(4,140) = 11.71, *p* < 0.001, *F*(4,136) = 13.22, *p* < 0.001, respectively). On the STAI-S, these differences were observed between baseline (48.4 ± 27.1, high anxiety) and week 3 (41.7 ± 26.8, moderate anxiety, 95% CI [1.90, 11.55]), week 6 (37.1 ± 26.8, no/low anxiety, 95% CI [4.96, 17.65]), week 16 (37.9 ± 26.7, no/low anxiety, 95% CI [2.49, 18.45]), and week 32 (37.2 ± 25.9, no/low anxiety, 95% CI [4.58, 17.93]) (Bonferroni corrected *p* < 0.05 for all comparisons with baseline), with no differences in symptoms between any other time points.

With regard to symptoms of perceived stress, compared with baseline (29.0 ± 5.7), women reported significant reductions at week 6 (19.7 ± 5.3, 95% CI [1.33, 7.07]), week 16 (19.2 ± 5.7, 95% CI [1.87, 7.55]), and week 32 (19.1 ± 6.1, 95% CI [2.12, 7.54]) (all Bonferroni corrected p < 0.001). Furthermore, compared with week 3 (22.4 ± 5.2), mothers experienced significant reductions in their symptoms of stress at weeks 6 (95% CI [0.91, 4.46]), 16 (95% CI [0.47, 5.93]), and 32 (95% CI [0.33, 6.30]) (all Bonferroni corrected *p* < 0.05), altogether indicating that the effect of the intervention only became evident after week 3.

##### Social support and loneliness

To ascertain whether online singing improved perceived social support and self-efficacy, and reduced loneliness, we compared total scores on the ONS (Fig. 4), the UCLA loneliness scale, the GSE-6, and the MSPSS, respectively, across the five study time points.

Mothers reported significant changes on the ONS between baseline and week 6, whereby they experienced an increase in their life satisfaction (50.5 ± 23.0 to 72.8 ± 11.7, *F*(4,44) = 5.28, *p* = 0.001, 95% CI [− 39.86, − 4.64], Bonferroni corrected for baseline to week 6 *p* = 0.01) and an increase in their feelings of worthwhile (51.7 ± 30.4 to 78.6 ± 15.1, *F*(4,40) = 3.15, *p* = 0.024, 95% CI [− 52.79, − 0.85]. Bonferroni corrected for baseline to week 6 *p* = 0.041). There was no significant change in their overall happiness or sense of anxiety.

There were no significant differences in scores across time on the MSPSS, GSE-6, and UCLA Loneliness scale regarding perceived support, self-efficacy, and loneliness (see Supplementary Fig. 1).

##### Exploratory analyses

To explore whether reduction in mothers’ EPDS scores over the course of the study was associated with within-participant change across other dimensions of mental health (depression on BDI, anxiety on STAI-S, perceived stress on PSS), we conducted a fixed-effects regression.

We found that the regression model overall was statistically significant, suggesting an association between EPDS reduction and other psychiatric symptom reduction (*R*^2^ = 0.613, *F*(38,170) = 7.09, *p* < 0.001). Specifically, within participants, a reduction in depressive symptoms on the EPDS was associated with a decrease in other aspects of depressive symptoms on the BDI (*β* = 0.369, *p* = 0.009, 95% CI [0.045,0.318]) and with anxious symptoms on the STAI-S (*β* = 0.353, *p* = 0.021, 95% CI [0.012,0.146]). Interestingly, reduction of depressive symptoms on the EPDS was not associated with reduction in feelings of stress on the PSS.

Next, to explore whether certain clinical and sociodemographic variables may have been associated with treatment response, we conducted univariate correlations (see Table 2) between the change in EPDS scores (baseline to week 6, delta EPDS) and:

- Variables that are risk factors for postnatal depression, according to previous literature: history of childhood maltreatment, adult experience of intimate partner violence, stressful life events in the perinatal period and throughout life, complications in pregnancy and/or delivery, problems with feeding the baby, and prior and/or current episodes of depression at baseline [44–46].
- Treatment-related variables that can influence the outcome: baseline EPDS score, number of singing sessions attended, and concurrent attendance of other mother–baby groups.

**Table 2:** Univariate correlations between demographics and risk factors for postnatal depression and change in EPDS score between the end of intervention and start of intervention (EPDS delta)

	**EPDS delta**
History of childhood maltreatment^a^	− .39*
Types of maltreatment:	
Physical abuse	− .17
Sexual abuse	− .19
Antipathy	− .35*
Neglect	− .38*
Number of types of maltreatment^b^	− .58*
Adult experience of intimate partner violence^a^	− .38*
Physical abuse	− .16
Sexual abuse	− .01
Psychological abuse	− .38*
Threatening life events in the perinatal period^a^	− .27
Intrusive life events throughout life^a^	0.11
Pregnancy and/or childbirth complications^a^	0.02
Feeding complications^a^	0.31
Prior major depressive disorder episode^a^	0.13
Current major depressive disorder episode on SCID at baseline^a^	0.08
EPDS score at baseline^b^	0.16
Attendance of other mother–baby activity group^a^	0.06
Number of singing sessions attended^b^	.35*

^a^Point-Biserial correlation coefficients are presented

^b^Pearson’s correlation coefficients are presented

^*^p < .05; **p < .01

Variables that were negatively correlated with treatment response (EPDS delta) were a history of childhood maltreatment (specifically antipathy and neglect) and adult experience of intimate partner violence (specifically psychological abuse), suggesting that these were associated with a smaller response to intervention. Furthermore, the mean number of singing sessions was positively correlated with EPDS delta, indicating, unsurprisingly, that those who attended more sessions had greater benefits to their mental health. Of note is that a previous history of depression, a clinical diagnosis of MDD on the SCID, or the baseline EPDS scores, were *not* associated with treatment response.

Finally, we conducted a regression to understand the independent or synergistic roles of childhood maltreatment, adult intimate partner violence, and other sociodemographic and clinical factors, in predicting treatment response. As history of childhood maltreatment and adult experience of intimate partner violence were not correlated (*r* = 0.26, *p* = 0.13), both variables were entered into the regression.

In an unadjusted model, we found that the presence of childhood maltreatment was significantly associated with the EPDS delta and accounted for 15% of the variance (*p* = 0.028, 95% CI [− 6.49, − 0.40]). Presence of adult intimate partner violence was not significantly associated with EPDS delta and thus did not further predict treatment response on top of childhood maltreatment, and, altogether, the total model explained 16% of the variance in EPDS delta (see Table 3). We next added sociodemographic and clinical factors to the model (maternal ethnicity, household income, problems in the pregnancy/delivery, problems feeding the baby, attendance of other mother–baby groups, and number of singing sessions attended) and found that with the addition of those factors, childhood maltreatment was still significantly predictive of treatment response (*p* = 0.017, 95% CI [− 8.45, − 0.93]). Finally, we added risk factors for PND to the model (threatening life events, intrusive life events, past MDD, current MDD, and baseline EPDS) and found that upon addition of these final factors, childhood maltreatment remained marginally significantly predictive of EPDS delta (*p* = 0.057, 95% CI [− 9.29, 0.14]), suggesting that, overall, childhood maltreatment was the largest factor contributing to treatment response, due to its presence as a risk factor for developing lifetime or postnatal depressive symptoms.

**Table 3:** Analysis of factors predictive of treatment response (EPDS delta)

Predictors	Coefficient	95% CI	*p*	*R*^2^
Unadjusted model with CM and IPV				
CM^a^	− 3.25	− 6.38 to − 0.11	.028	.15
IPV^a^	2.00	− 4.32 to 8.32	.523	.16
Adjusted for demographics^b^				
CM	− 4.69	− 8.45 to − 0.93	.017	–
IPV	0.10	− 3.50 to 3.52	.996	–
Adjusted for demographics and other risk factors for PND^c^				
CM	− 4.57	− 9.29 to 0.14	*.057*	–
IPV	− 1.81	− 6.45 to 2.82	.422	–

^a^Both entered simultaneously into model

^b^Demographics include maternal ethnicity, household income, physical problems with pregnancy/delivery, problems feeding baby, attendance of other mother–baby groups, number of sessions attended

^c^Risk factors for PND include threatening life events, intrusive life events, prior MDD, current MDD, EPDS at baseline

## Discussion

SHAPER-PNDO is the first study to our knowledge to investigate the feasibility and clinical efficacy of online group singing sessions for new mothers with PND. This pilot study was an online adaptation of the community M4M singing intervention for PND, which was rapidly developed because of the 2021 COVID-19 lockdowns in the UK, a time of extreme social isolation and increased mental health vulnerability. Although this was a single-arm clinical study (without a control arm), we produced data indicating that the completion of our 6-week online M4M intervention is associated with a significant decrease in postnatal depressive, anxious, and stress symptoms, and a significant increase in sense of well-being, with results maintained at least 6 months following the end of the intervention.

The intervention was found to be feasible in terms of retention and completion, with a high attendance to sessions and a good level of completion of study visits and assessments; in fact, when compared with other online interventions used for depression (such as internet-based CBT), our intervention had a comparable or higher attendance and retention rate, reflecting the clinical benefits and enjoyment mothers got out of the group singing sessions [47, 48]. Given the societal context of the study, we believe that the COVID-19 pandemic worked as both a facilitator and a barrier to recruitment: while mothers reported a need for social interaction via online platforms, “online fatigue” due to the vast majority of social interaction in that period being conducted online may have hindered recruitment to the study, which was lower than planned. Furthermore, we decided to interrupt the study as soon as lockdown restrictions were lifted, in order to return to the face-to-face delivery of the intervention. Despite a lower recruitment than expected, the retention and study completion within our sample points to the feasibility of conducting a larger RCT, though efforts will need to be made to broaden recruitment. Specifically, recruitment will need to be conducted in wider venues than solely social media and primary care, for example, via health visitors, mental health clinicians, at baby-weigh clinics and children’s community centres.

Contrary to previous literature reporting sociodemographic risk factors for PND [49], our sample was relatively homogeneous (as discussed in our limitations below), with the vast majority being older, white, highly educated, married/cohabiting, and employed or on maternity leave. It is, however, noteworthy to highlight that a considerable proportion of women reported a childhood history of maltreatment (36%) and/or a past/presence of intimate partner violence (41%), consistent with literature reporting associations between maternal abuse and the development of postnatal depression [46, 50]. Additionally, many mothers reported intrusive life events, mostly bullying, and some reported injury, assault, or death of a relative or a friend, which is consistent with high COVID-19 infections in this period, as mentioned by participants during the baseline interview. Around 75% of participants had a history of MDD, a high-risk factor for developing PND [4].

Our secondary outcome was met: mothers experienced a significant reduction in symptoms of PND (EPDS) between the start and end (6 weeks) of the online intervention, and reassuringly, this reduction was sustained at all follow-up points in the study, up to 6 months post-treatment. These results indicate that only 6 weeks of online M4M intervention may be needed for effective and sustained alleviation of postnatal depressive symptoms. This is consistent with findings in the initial in-person three-arm RCT of M4M, which found EPDS decreases by week 6 of intervention [24]. Most importantly, online M4M is a promising treatment for PND for populations experiencing social isolation or other barriers to in-person treatment. These findings are especially pertinent, as a recent review discussed the benefits of partaking in online psychological interventions for pregnant women’s mental health during the COVID-19 pandemic [51]. Moreover, given that symptoms reduced already by week 3, and even further so by week 6, it is evident that our intervention alleviates depressive symptoms very quickly when compared with other remote interventions for PND which take up to 12 weeks [48]. Regarding the HDRS, a clinician-administered measure of severity of depression, the reduction in score translates to a change from mild-moderate depression at baseline to complete remission of symptoms of depression at week 6. These findings are more pronounced than those of the EPDS (at week 6 there is, on average, still mild depression); however, the EPDS is highly sensitive and specific for perinatal depressive symptoms, while the HDRS is a broad-spectrum instrument for depression severity. Overall, this suggests that, while women may have still had some lingering symptoms of depression, their symptoms were no longer clinically significant.

In addition to PND symptoms, women also experienced significant decreases in anxiety and stress levels. The reductions happened as early as week 3 of intervention which were concurrent to the reductions in EPDS score, and this effect was also sustained throughout the intervention and follow-up period. The reduction in tandem at week 3 suggests that the online M4M intervention has a holistic action in improving mood and stress. This is an important finding given that anxiety and stress are often [52] and that other online interventions such as CBT are not able to successfully attenuate both depressive and anxious symptoms simultaneously [48, 53].

Our intervention also improved self-reported aspects of well-being, namely life satisfaction and the worthwhileness of life aspects (on the ONS), perhaps due to a sense of routine and purpose, and the impact of a weekly activity to look forward to during COVID lockdowns when other activities were not available. As previous studies examining the effects of maternal singing on well-being have also found similar improvements [54], it is an encouraging finding that an online intervention is just as efficacious as the face-to-face singing experience. Unfortunately, we did not find an effect on (increasing) social support or (reducing) feelings of loneliness. However, due to the unprecedented social isolation that the lockdowns imposed on new mothers, from other mothers, friends and family, and health services, it is perhaps unsurprising that the online intervention would not be effective enough on these dimensions.

Regarding factors that could have been associated with treatment response, we found no effect of a history of MDD, which most women in the study had, a clinical diagnosis of MDD on the SCID, or the baseline EPDS score. Finally, risk factors concurrent to mothers’ PND, including stressful life events, also did not seem to impede upon the effect of online M4M. This is reassuring and suggests that the intervention was no less effective for those who had more severe, complex, or longstanding histories of depression. It is especially important to provide an intervention suitable for all degrees of depression, given that the care pathway for PND currently varies based on illness [55]. Furthermore, we found that attendance of other mother–baby activities was not associated with decrease in EPDS score, suggesting an effect of the M4M online intervention in reducing symptoms of PND over and above other activities.

When considering factors that *did* impact response to treatment, we found that childhood maltreatment and intimate partner violence were both negatively associated with change in EPDS score, such that mothers who presented with either of these risk factors experienced a smaller reduction in their depression, although our hierarchical regression showed that presence of childhood maltreatment was the most important predictor. Previous studies have found that adults who experienced childhood maltreatment are more likely to experience depression in adulthood, especially in the perinatal period [50, 56], and are less likely to respond to standardised treatment for their depression or more likely to experience a slower recovery [57, 58]. This is thought to be the case due to the often-complex nature of depression related to childhood abuse, in that the perinatal period—a time of intense hormonal [59] and emotional readying to parent—may reactivate trauma of negative maternal representations from [60]. Furthermore, studies that have examined childhood and adulthood abuse have found that abuse specifically endured in childhood is predictive of a more difficult-to-treat perinatal depression [60], a finding that we replicate in our study.

In terms of limitations, the sample’s homogeneity is a potential issue, as it did not represent the socio-economic diversity in the UK (although the rates of childhood and adulthood abuse were remarkably high). Future efforts should be undertaken to modify the intervention to ensure it is inclusive and addresses barriers to treatment, especially given that barriers to treatment are more likely to impact socioeconomically disadvantaged groups. Of note, though, is that due to the COVID-19 pandemic lockdown, usual routes of recruitment (e.g. GP surgeries, children and family centres, baby weigh clinics) were not possible. The study was, however, feasible for the sample recruited, as well as clinically effective. Another limitation to our study was that we did not collect data on the implementation effectiveness of our online M4M intervention or collect feasibility and acceptability data according to a data collection framework such as the Theoretical Framework of Acceptability [61] or the Reach, Effectiveness, Adoption, Implementation, and Maintenance framework [62]. This was due to limited resources, how quickly we adapted and began our intervention to help new mothers who were profoundly suffering as a consequence of the COVID-19 lockdowns, and the fact that we did not want to overburden mothers who were in a particularly vulnerable time with extensive and additional questionnaires.

Furthermore, given the study design of a non-blinded single-arm clinical study (without a control arm), it is therefore not possible to infer, with certainty, whether the results observed are solely due to the effectiveness of this intervention or, moreover, whether the knowledge of attending the singing sessions influenced the outcomes. However, it is notable that the changes in mental health and well-being were found exclusively during the period of intervention, with maintenance rather than continued recovery following. This suggests a potential causal effect of the intervention. Finally, our simple size was quite limited due to difficulties in recruiting during the COVID-19 lockdown.

To further understand how singing interventions work, our group is currently conducting a full-scale, placebo-controlled, randomised study of the face-to-face M4M, SHAPER-PND (NCT04834622), to ensure that reductions in PND can be attributed directly to the M4M intervention, and to investigate the psychosocial and biological mechanisms underpinning the putative improvement. Furthermore, in order to better understand whether our online M4M intervention can effectively reach and treat new mothers experiencing barriers to care and, moreover, to understand the mechanism behind symptom reduction in our online intervention, we are in the process of seeking funding to translate this pilot study into a full-scale RCT. According to the criteria for pilot trial progression into full RCTs, set by Avery et al. [28], we therefore consider that we are at the amber-light stage: though our overall recruitment to the pilot study was quite below target, we do believe this aspect of feasibility is rectifiable in a larger-scale RCT with revised recruitment strategies, and, furthermore, given that our pilot study shows promising evidence for other aspects of feasibility, in addition to the clinical and well-being effectiveness of online M4M for postnatal depression, our pilot study merits progression to an RCT.

## Conclusions

Our single-arm study provides evidence of feasibility and preliminary antidepressant efficacy of a new, online singing intervention for PND. While replication of our study is needed, we propose that this intervention could be helpful beyond times of mandatory social isolation, in women in the perinatal period who cannot access face-to-face interventions for health or social reasons or live in areas that do not have such interventions.

## Supplementary Information

**Additional file 1: Supplementary Figure 1.** Mean MSPSS, GSE, and UCLA scores at baseline, week 3, week 6, week 16, and week 32.

## Data Availability

The data that support the findings of this study are available from the corresponding author, RB, upon reasonable request.
