# Supplementary figures and images for: Feasibility, clinical efficacy, and well-being outcomes of an online singing intervention for postnatal depression in the UK: SHAPER-PNDO, a single-arm clinical trial
Source: Pilot Feasibility Stud. 2023 Jul 27;9:131. doi: 10.1186/s40814-023-01360-9 (PMC10373337; doi:10.1186/s40814-023-01360-9)

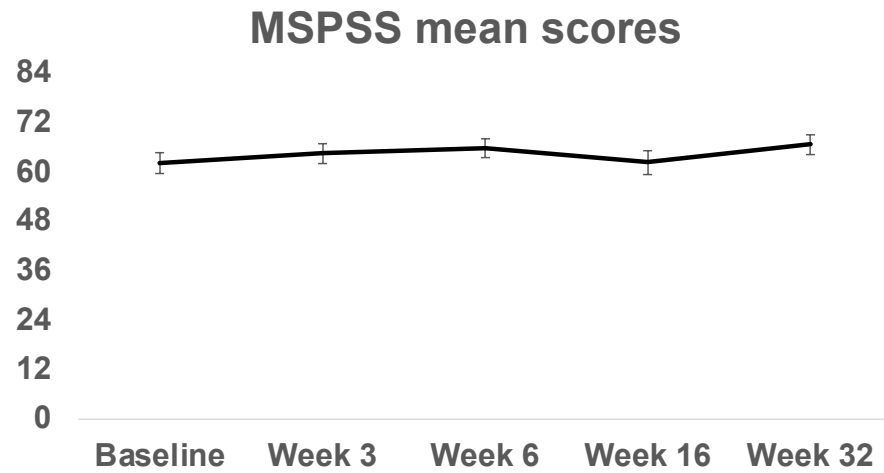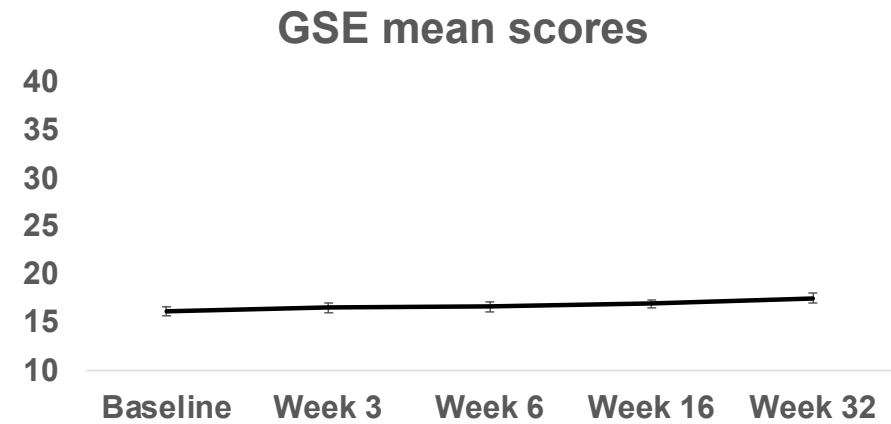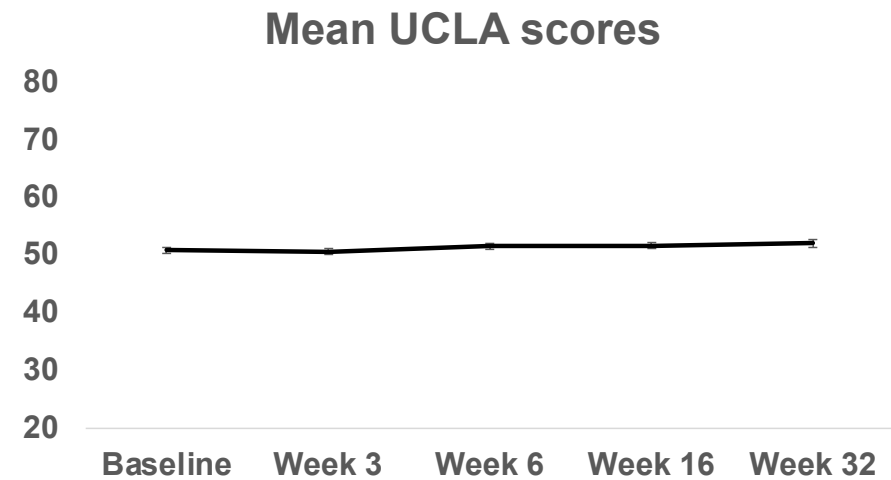

**Supplementary Figure 1.** Mean MSPSS, GSE, and UCLA scores at baseline, week 3, week 6, week 16, and week 32

Supplement: Supplementary file 1 — Additional file 1: Supplementary Figure 1. Mean MSPSS, GSE, and UCLA scores at baseline, week 3, week 6, week 16, and week 32. [file 40814_2023_1360_MOESM1_ESM.pdf]
